# Research and Experimental Testing of a Remotely Controlled Ankle Rehabilitation Exoskeleton Prototype

**DOI:** 10.3390/s25216784

**Published:** 2025-11-06

**Authors:** Assylbek Ozhiken, Gani Sergazin, Kassymbek Ozhikenov, Haohan Wang, Nursultan Zhetenbayev, Gulzhamal Tursunbayeva, Asset Nurmangaliyev, Arman Uzbekbayev

**Affiliations:** 1 Institute of Mechanics and Engineering named after Academician U.A. Joldasbekov, Almaty 050010, Kazakhstan; ozhiken11@gmail.com; 2 School of Information Sciences, University of Illinois at Urbana-Champaign, Champaign, IL 61820, USA; 3 ALT University, Almaty 050013, Kazakhstan; 4 Department of Robotics and Technical Tools of Automation, Satbayev University, Almaty 050013, Kazakhstan; 5 Department of Information Security, Eurasian National University, Astana 10000, Kazakhstan; 6 Department of Information Technologies, “Q” University, Almaty 050026, Kazakhstan; 7 Research Institute of Applied Science and Technologies, Almaty 050013, Kazakhstan

**Keywords:** lower limb rehabilitation, remote control system, exoskeleton, ankle joint, remote control device, motion biomechanics, wireless communication platform

## Abstract

Today, there is a high demand for remote rehabilitation using mobile robotic complexes all over the world. They offer a wide range of options for convenient and effective therapy at home to patients and the elderly, especially those bedridden after musculoskeletal injuries. In this case, modern approaches to the development of exoskeletons for the rehabilitation of the lower extremities are especially relevant for the effective restoration of lost motor functions. Taking into account the advantages and features of robotic rehabilitation, this work is devoted to the development of a prototype exoskeleton for the ankle joint and experimental studies of the remote control module. The proposed new exoskeleton prototype design was integrated with a mobile wireless communication platform, allowing remote control of the position of the exoskeleton foot using a remote control device. As a result of functional testing, the root mean square error (RMSE) was 23.9° for dorsiflexion/plantarflexion movements and 12.8° for inversion and eversion movements, as well as an average signal transmission delay of about 100 ms and packet loss of 0.6%. These results reflect the technical feasibility of remote control at a distance of up to 10 m. The developed system is mobile, autonomous, and easy to use, which confirms its suitability as a laboratory platform for functional verification and testing of module consistency.

## 1. Introduction

Ensuring universal health coverage for all segments of the population and reducing mortality from non-communicable diseases through prevention and treatment is a priority of the United Nations (UN) Agenda for Sustainable Development Goals (SDGs) [1,2]. This is achieved by providing access to essential health services without financial hardship. These include health promotion, prevention, treatment, including palliative care, and rehabilitation [3]. They must be of high quality, effective, safe, and affordable to provide access to every patient in need.

Today, an aging population and an increase in the number of people suffering from chronic diseases, including alarmingly rising mortality rates from cardiovascular diseases, are placing an increasing burden on healthcare systems around the world. These trends highlight the need to find new, technologically effective solutions aimed at ensuring timely rehabilitation for the population. This is especially true in cases where these diseases result in loss of motor function, neurological disorders, and musculoskeletal disorders.

According to data from [4] the Global Burden of Disease study, stroke ranks among the leading causes of long-term disability and loss of motor function, second only to ischemic heart disease. According to statistics [5], more than 17.9 million people die from cardiovascular disease each year, with more than four out of five deaths related to heart attack or stroke, and about one-third of these cases are premature deaths. Surviving patients may develop consequences such as hemiparesis, hemiplegia, speech and communication disorders, cognitive disorders, including partial or complete loss of motor function [6]. Muscle disorders controlling movement in the ankle joint due to neuromuscular disorders are also common [7,8]. This significantly affects the biomechanics of walking and leads to a decrease in walking speed, as well as changes in gait kinematics and an increased risk of falls. In this case, effective restoration of lower limb function is a key stage in the rehabilitation of stroke patients, requiring an individualized approach.

In modern conditions, the most common method of rehabilitation for patients after stroke and musculoskeletal injuries is carried out using innovative wearable and robotic devices. They are aimed at restoring lost motor functions and increasing the effectiveness of therapeutic procedures. However, most of them are bulky and primarily intended for clinical use, which limits the possibilities for home rehabilitation of patients. In addition, inpatient rehabilitation therapy requires frequent visits to medical facilities for continuous monitoring of patients’ condition and remains costly due to its labor intensity and the need for constant involvement of physiotherapists [9]. These problems highlight the need to create affordable, lightweight, and remotely controlled robotic wearable devices that accelerate the recovery of lower limb function in patients with musculoskeletal injuries and stroke survivors, as well as improve the condition of bedridden patients with limited mobility.

Given the limitations outlined above, this study aims to create innovative solutions in the field of remotely controlled exoskeletons for the rehabilitation of the lower extremities. The proposed system integrates a lightweight prototype ankle exoskeleton with two degrees of mobility (2-DoF) and a wireless control platform based on IMU and Bluetooth, providing smooth and consistent foot movements in the sagittal (dorsal/plantar flexion) and transverse (inversion/eversion) planes at a distance of up to 10 m. Functional tests were carried out in laboratory conditions without human intervention for the evaluation of synchronization, control accuracy, and stability of wireless communication. The architecture of the system confirms the ability to implement wireless control in real time and safely reproduce movements within the established physiological limitations. Unlike existing systems, the proposed design includes biomechanical RoM safety limits that prevent joint overloading. Self-locking screw mechanism provides mechanical safety without complex torque sensors. The low-budget laboratory prototype is controlled via a Bluetooth connection that does not require an external PC. At this stage, the system is a laboratory proof of concept for remote ankle rehabilitation. The device has been developed with potential use for individuals with mobility impairments, pending future clinical validation.

The article consists of five sections and is organized as follows: The first section provides an overview of existing problems, relevance, and research objectives. The second section contains a brief analysis of existing solutions in the field of lower limb exoskeleton development and remote control systems. The basic architecture of the mobile remote control system, hardware and software components, control algorithms, and the process of creating a prototype ankle joint exoskeleton, including safety aspects, are discussed in the third section on materials and methods. The next section includes functional testing of the exoskeleton prototype in the sagittal (*X*-axis) and transverse (*Y*-axis) planes, including verification of the mobile wireless communication platform’s performance. The results are summarized, and the engineering prospects of the system are outlined, clinical application will require future validation.

## 2. Related Work

Modern robotic rehabilitation technologies for the lower limbs are focused on restoring and maintaining mobility in people with motor impairments. Researchers around the world have proposed a wide range of wearable devices [10] designed for rehabilitation. Among them, exoskeletons for the entire lower limb [11], ankle joint [12], knee [13] or hip [14], with varying degrees of freedom (DoF) [15], have found popular application. The use of such systems contributes to improving the effectiveness of rehabilitation and the quality of life of patients with limited mobility who have suffered a stroke or musculoskeletal injuries. They are mainly applicable in early rehabilitation processes to improve blood circulation, including supporting muscle activity to help prevent atrophy [16]. They are also often used in the process of restoring neuroplasticity in people after musculoskeletal injuries, performing passive range of motion (PRoM) exercises for paralyzed limbs [17].

One of the first commercially available lower limb rehabilitation systems was developed by the Swiss company Hocoma [18]. The Lokomat robotic exoskeleton, integrated with a treadmill and partial weight support system, is widely used in clinical practice. It is particularly often used for the rehabilitation of patients with spinal cord injuries, traumatic brain injuries, stroke, or neurological diseases [19]. Despite its proven effectiveness in numerous clinical studies, the Lokomat remains a bulky (1000 kg (2204 lbs)) and expensive stationary device. It is not suitable for individual home rehabilitation or telerehabilitation with remote control and monitoring. Another promising solution in this area was proposed by researchers at the Massachusetts Institute of Technology (MIT) [20]. As a rigid exoskeleton, Anklebot was originally developed for ankle joint rehabilitation after a stroke. Later, the system was adapted for use with children with neurological disorders. It is based on theories of motor learning, neuroplasticity, and dynamic systems. This allows patients to perform a large number of repetitions of targeted movements in a seated position with two degrees of freedom. However, despite its successful use in clinical practice, AnkleBot remains a stationary, heavy (3.5–4 kg) device that is expensive and complex in design. These features make it unsuitable for use in a personalized home environment.

Clinical and home use combine the ReWalk wearable exoskeleton [21], designed to restore gait in spinal cord injuries. Despite its effectiveness, the device remains heavy (20–25 kg), requires crutch support, has a limited speed range, and is expensive, which reduces its accessibility for widespread use.

Many similar studies have been devoted to the development of mobile lower limb exoskeletons [22]. They are mainly designed to facilitate regular physiotherapy for patients who have suffered a stroke and have lower limb dysfunction. In this area, the authors [23] developed an exoskeleton for knee joint rehabilitation. The system combines axial bone loading and auxiliary torque to support gait. The modular cable architecture allowed the authors to reduce the weight of the wearable part by 30–40%, thereby increasing the efficiency of force transmission (up to 96%). The presented approach, when confirmed by clinical validation, is promising for post-operative rehabilitation of fractures and restoration of muscle function at home. Another group of authors [24] designed a single-leg rehabilitation exoskeleton suitable for home use. Precise control of motor position and torque in the lower limb joints is achieved by a PID controller. However, a wireless control strategy based on a PID controller involves complex integration of sensor networks, which complicates real-time data analysis. A new design for a 6 DoF lower limb exoskeleton platform using Bowden cables was developed by the authors [25]. The inexpensive, accessible platform has a split remote drive and interface with a low-level architecture remote control system. Despite its promising prospects, the exoskeleton system remains heavy (≈10 kg) and requires further thorough testing in real-world conditions to confirm the high performance of the system in positioning and torque acceleration modes. In their article, the authors [26] successfully demonstrated the improved Remotion medical exoskeleton. The developed intelligent remote control system, based on a Wi-Fi connection between a PC and the exoskeleton, enables the activation and adjustment of modes with a functional electrostimulation system and electromyography channels. The limitations include the heavyweight of the exoskeleton (about 15 kg) and the complexity of the dynamic model of human gait during remote control.

Also, of interest are works proposing a 4DOF lower limb robot [27], a 2DOF lower limb exoskeleton design [28], including the development of a self-tuning subordinate control system based on a neural network for nonlinear electric drives of the lower limbs of the exoskeleton [29]. Several studies investigated the development of rehabilitation devices [30], hybrid systems [31], and lower limb exoskeletons [32] designed to treat lower limb weakness in acute and subacute ischemic stroke. They are applicable to patients who have suffered a stroke. The technical assessment in the listed works, clinical feasibility, and results of experimental studies emphasize the need for regular physiotherapy to improve the condition of patients with lower limb disorders. The importance of developing a concept for home rehabilitation of patients using innovative robotic exoskeletons is also noted [33]. Taking into account all the features of lower limb exoskeletons, Table 1 presents the main characteristics of the devices, including weight, range of motion, torque, control methods, and application features.

Analysis of Table 1 shows that most existing exoskeleton systems achieve high values of torque and control accuracy, but this is accompanied by an increase in the mass, complexity and cost of the device. For example, the Lokomat and ReWalk systems provide a full walking cycle and high torque (40–60 Nm), but remain bulky (20–1000 kg) and are designed exclusively for stationary use. At the same time, lightweight solutions such as AnkleBot (3.5–4 kg) are distinguished by high control accuracy, but require rigid structures and fixed installation, which limits the possibilities of use at home.

Regular home rehabilitation is becoming even more effective thanks to the development of mobile lower limb exoskeletons with autonomous [34,35] and decentralized [36] control systems. These developments open up new opportunities for improving personalized rehabilitation, helping to reduce the burden on medical staff. Adhering to the basic criteria [37] of the concept of safe physical [38] interaction between humans and robots [39], numerous studies [40,41,42,43] have proposed the design [44] of an exoskeleton for stabilizing [45] nonlinear systems with a high degree of freedom. The presented developments provide the possibility of effective rehabilitation of the lower limbs in various conditions, without disrupting the continuity of training programmers.

All of the above solutions are aimed at supporting the healthcare system and integrating rehabilitation into all levels of healthcare. A review of existing research has demonstrated a number of key features characteristic of exoskeleton systems. A number of important criteria were noted, including system mobility, safety, biomechanical compatibility, and effective device control that does not require complex settings or technical skills.

Today, lower limb rehabilitation remains an underfunded area that is not given sufficient priority in many countries. Key barriers have been identified among the main problems in the recovery process, especially among stroke patients [46]. These include the remoteness of medical facilities, a shortage of qualified personnel, the financial inaccessibility of rehabilitation services, and the difficulty of ensuring continuous training due to logistical constraints [47,48]. Along with these barriers, the impact of the global COVID-19 pandemic, when hospitals, medical centers, and rehabilitation facilities were completely paralyzed, has also been highlighted [49]. As a result, many were deprived of the opportunity to receive the necessary rehabilitation care in a timely manner. This exacerbated the consequences of musculoskeletal disorders and slowed down the recovery process for patients [50]. These factors underscore the need to develop affordable robotic rehabilitation systems that combine stationary and remote control modes and are suitable for use in a variety of conditions, including periods of healthcare system instability and emergencies.

Adhering to this concept, this study focuses on the development of a mobile remote control system for a prototype ankle exoskeleton that combines flexibility, accessibility, and personalization of rehabilitation procedures in various rehabilitation settings.

Unlike existing analogs, the developed prototype ankle exoskeleton has functional mobility in 2 active DoF degrees of freedom. The main advantage of the developed system is its affordable, safe, and modular architecture, which provides laboratory testing of remote rehabilitation technologies at a distance of up to 10 m. With a weight of about 3 kg and a safe torque of 8–10 Nm, the device provides sufficient force for ankle rehabilitation, while remaining light and convenient to use. The use of a self-braking screw mechanism eliminates the need for torque sensors and increases passive safety when the power is turned off. The proposed system represents an optimal combination of required power characteristics and mobility. This approach provides a reliable basis for further experimental testing and subsequent clinical validation.

## 3. Materials and Methods

The ankle joint plays an important role in human movement [51]. Its functionality is determined by three basic movements of the foot relative to the three coordinate axes X, Y, and Z. Movements along the *X* axis form the movement of the foot towards the front (flexion) and back (extension) surfaces of the lower leg. The transverse *Y* axis forms the rotation of the inner (inversion) and outer (eversion) edges of the foot towards the surface. Movements along the vertical *Z* axis form inward and outward rotation. A detailed analysis of the biomechanical characteristics of the ankle joint is presented in the authors’ previous work [52], which resulted in the design, kinematic diagram and main characteristics of the control systems [53] of the new ankle joint exoskeleton. Based on the research conducted, a functional prototype of an ankle joint exoskeleton was developed [54], where the anatomy of the ankle joint, kinematic design, 3D modeling, and simulation analysis were studied in detail using SolidWorks Simulation Professional software (2021) and the Motion Simulation module. As a continuation of this work, an improved version of the exoskeleton was proposed [55], where the main biomechanical characteristics and conceptual design of the ankle joint prototype were examined in detail. Building on previous research, this work focuses on the development and laboratory evaluation of a mobile remote control system for an exoskeleton designed for ankle joint rehabilitation, aiming to assess its technical feasibility prior to clinical studies. The development stages include the design of a prototype exoskeleton with a remote control module, the development of a wireless communication platform, the organization of safety measures, and functional testing of the device in laboratory conditions.

### 3.1. Determination of Biomechanical Characteristics for the Ankle Joint

**Figure 1:**
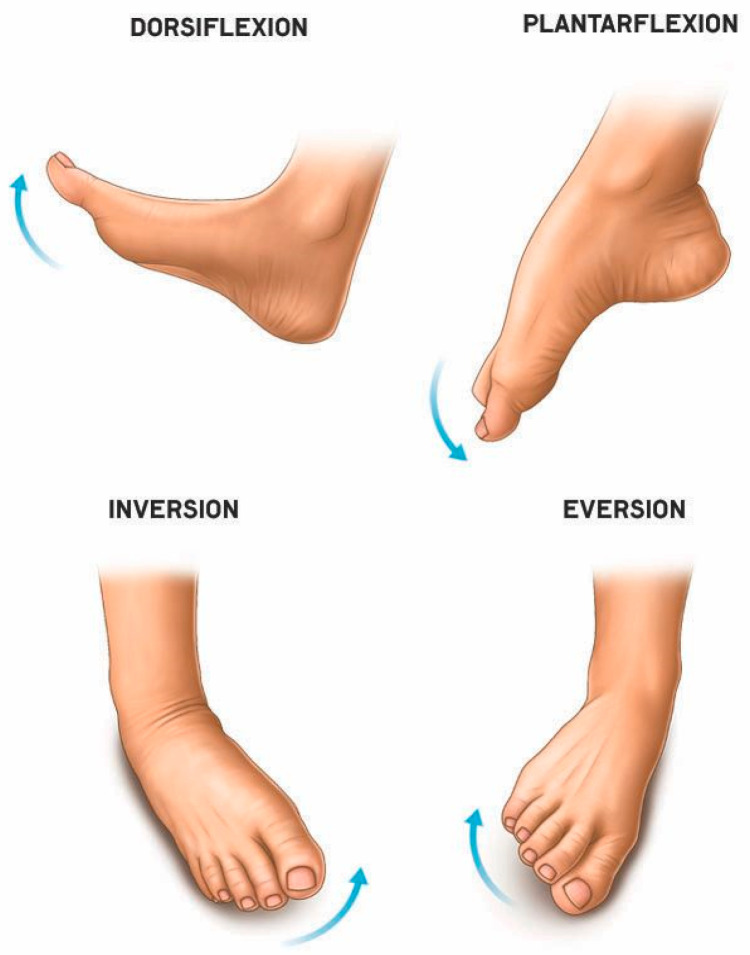
Diagram of foot mobility in the X and Y axes.

Taking into account the biomechanical characteristics of the ankle joint and its functional parameters, the initial phase of the experimental study considers movements along two axes (Figure 1), specifically the *X*-axis (Dorsiflexion/Plantar flexion) and the foot movement along the transverse *Y*-axis (inversion/eversion).

In a healthy state, the range of motion of plantar/femoral flexion can reach up to 71°. However, when walking on a flat surface, due to limitations in the range of motion of the ankle joint (RoM), it almost does not reach this entire range. The authors [56] noted that the actual mean average angle of inclination when walking on a flat surface under normal conditions is 30°, whereas in the elderly, this functional angle can be as low as 25.5°. In middle-aged people, the total range of motion in the transverse plane (abduction and adduction) normally reaches 55°, whereas in elderly people, this indicator decreases to an average of 32.5°. This small difference is explained by the fact that with age, involutional changes in the musculoskeletal system occur, including degenerative-dystrophic processes in joints, decreased elasticity of the ligamentous apparatus and age-related muscle hypotrophy. These physiological peculiarities of different age groups create the need to take into account the individual characteristics of the ankle joint and the age of the patient when carrying out rehabilitation measures. According to the data from the study [57], the maximum limits indicated in Table 2 are noted for experimental functional testing. The authors applied the Conical Joint Range of Motion Constraint method to determine RoM.

Considering the above maximum RoM limits, the basic ankle joint movements in the X and Y axes are the key parameters in the experimental study of the new remote-controlled ankle rehabilitation device in this paper.

### 3.2. Designing a Prototype Exoskeleton for Ankle Rehabilitation

**Figure 2:**
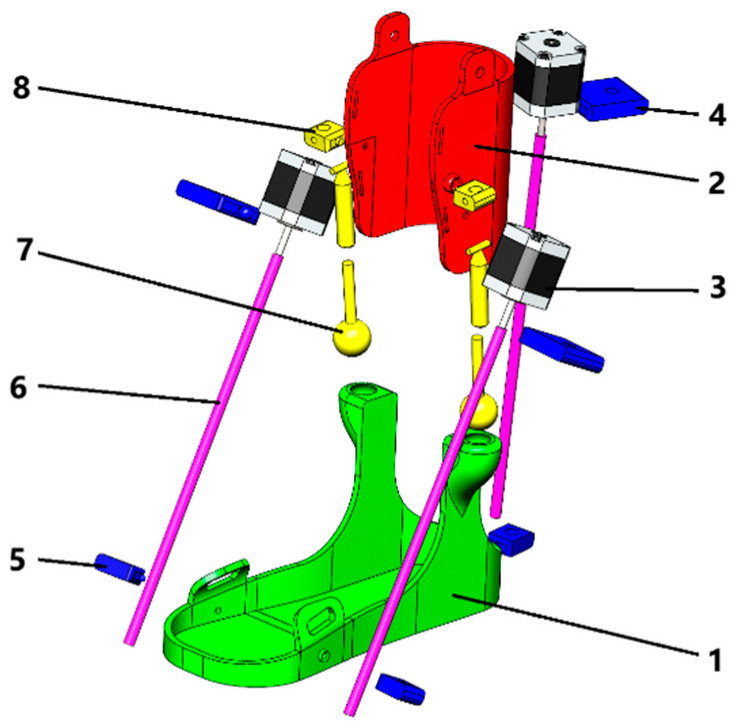
Exploded 3D model of rehabilitation exoskeleton [55]: 1—foot platform; 2—shank platform; 3—motor; 4—motor holder; 5—screw nut; 6—screw shaft; 7—ball joint; 8—cylindrical joint.

The design process of the prototype ankle exoskeleton began with the conceptualization phase, where the purpose and specific conditions for medical rehabilitation of the lower limbs were defined. SolidWorks Simulation Professional software and the Motion Simulation module were used to validate the data obtained and to create a virtual prototype. The results of virtual testing contributed to the creation of a 3D model of the exoskeleton prototype, which is presented in Figure 2.

**Figure 3:**
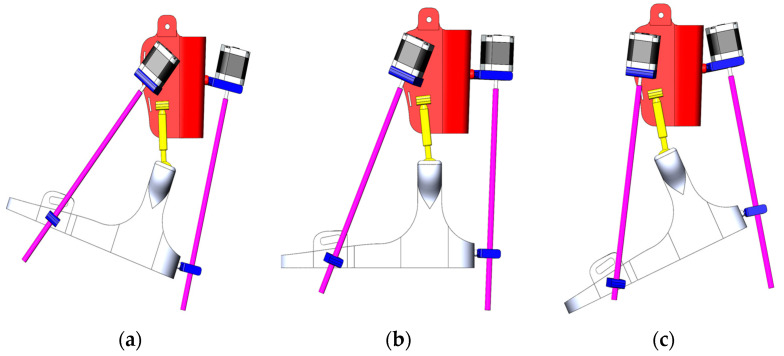
Exoskeleton positions in the sagittal plane: Dorsiflexion (**a**), Neutral position (**b**) and Plantar flexion (**c**).

**Figure 4:**
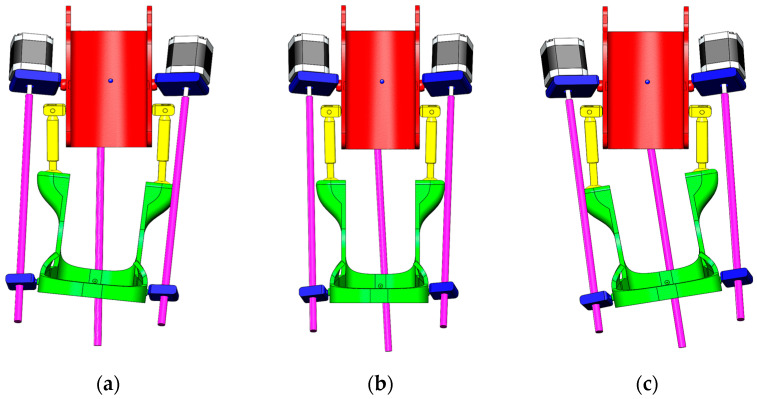
Ankle exoskeleton positions along the transverse plane: Eversion (**a**), Neutral (**b**), and Inversion (**c**).

Since the experimental study is conducted only in two planes—sagittal (dorsiflexion/plantar flexion) according to Figure 3 and transverse (inversion/eversion) according to Figure 4, movement along the vertical *Z*-axis (rotation) was excluded from the analysis at this stage.

At this stage of conceptualization of the prototype architecture, virtual functional testing of the 3D model was carried out, where the suitability of the mechanical system was realized. Next, a physical prototype was built, where numerical verification, mathematical modeling and simulation of the 3D model, and the operability of the exoskeleton control system were implemented. All the results obtained at a later stage could be easily integrated by their wireless communication platform for the remote control system of the exoskeleton prototype.

### 3.3. Developing a Wireless Communication Platform

**Figure 5:**
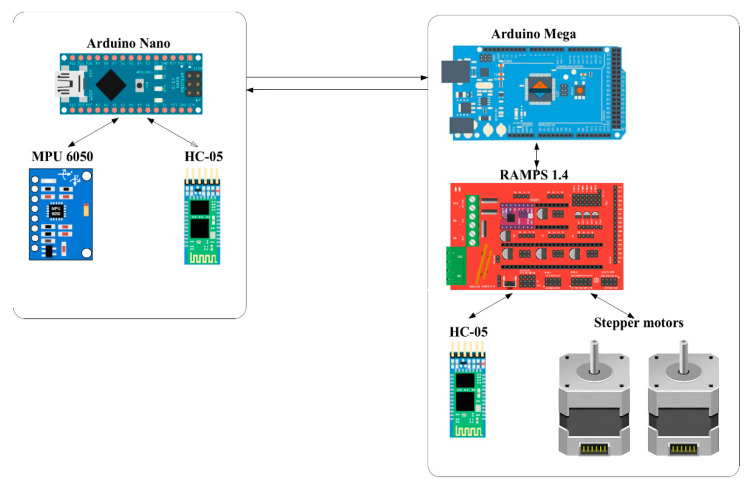
Architecture of wireless communication platform based on Bluetooth modules (HC-05).

The wireless communication platform should realize reliable transmission of control signals in real time. To ensure communication between the remote control device and the exoskeleton prototype, this system uses HC-05 module realizing Bluetooth connection. The architecture of the wireless communication platform shown in Figure 5 consists of two main components: the Remote control device unit and the actuator unit that drives the prototype. The Remote control device consists of an Arduino Nano controller, which has a connection to an MPU-6050 sensor that combines an accelerometer and a gyroscope to track the movements of the limb. The main purpose of this unit is to collect motion and orientation data for transmission via the HC-05 Bluetooth module. In this case, the HC-05 provides wireless communication between the Arduino Nano and Arduino Mega controllers. The operating frequency range of the HC-05 is 2.4 GHz with a typical communication radius of up to 10 m. The executive unit has a main Arduino Mega controller which, based on the received data from the Arduino Nano, controls NEMA 17 stepper motors via the RAMPS 1.4 expansion board. The second module, HC-05, is designed to receive data from the exoskeleton motion control unit.

**Figure 6:**
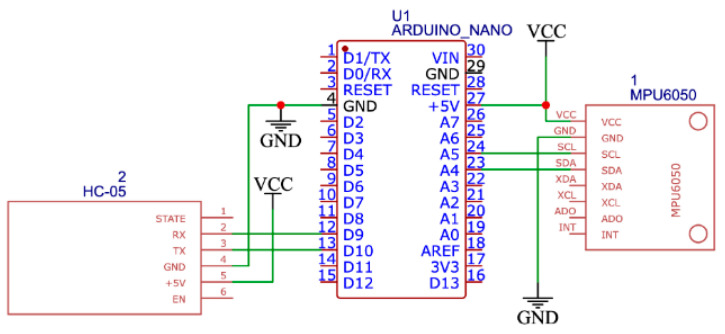
Electrical connection diagram of the Remote control device unit.

**Figure 7:**
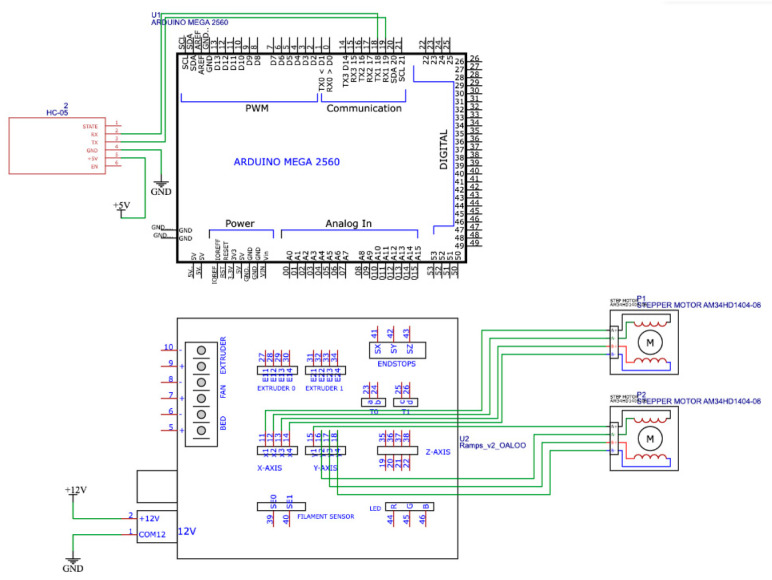
Electrical connection diagram of the executive unit.

The division of the system into two independent units allowed for modularity, flexibility in development and convenience in testing. The use of Bluetooth connection eliminates the need for wired interfaces, which is especially important when creating wearable devices. The wiring diagrams of the main components of the wireless communication platform are shown in Figure 6 and Figure 7.

### 3.4. Development of a Remote Control System for the Exoskeleton Prototype

**Figure 8:**
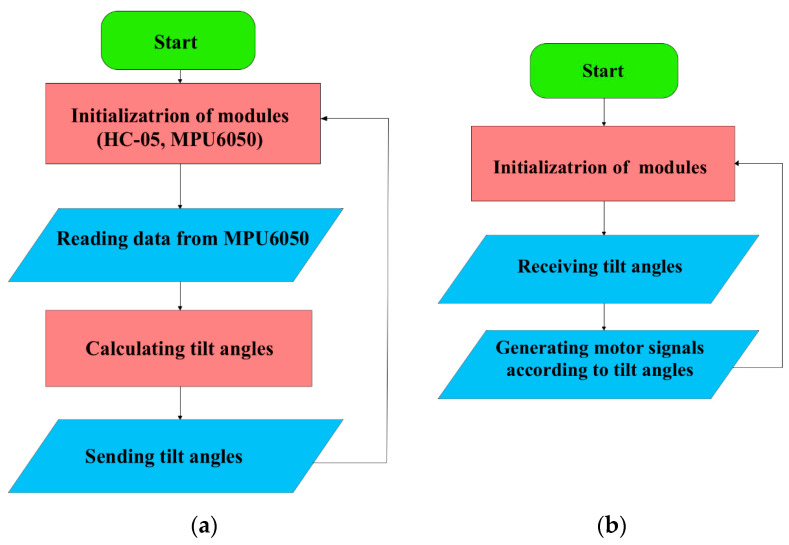
Exoskeleton remote control system: transmitter—Remote control device (**a**), receiver—actuator unit that drives the exoskeleton prototype (**b**).

The control system of the exoskeleton operates as a two-level transmitter–receiver architecture (Figure 8). After initialization, the MPU-6050 sensor reads ankle inclination data in the sagittal and transverse planes and sends them in real time to the actuator unit via the Bluetooth communication link described in Section 3.3. The actuator microcontroller converts the received angles into control signals for the stepper motors integrated into the screw mechanism of the exoskeleton.

The presence of a self-locking effect is the main advantage of the screw actuators, which prevents sudden jerks, smoothly converting rotational movements into linear ones. This allows the exoskeleton to hold the limb in position without the continuous use of a stepper motor, which facilitates rehabilitation and reduces the load on the drives. High accuracy and stable operation of the screw mechanism at low power consumption reduce the risk of re-injury and promote effective patient recovery.

### 3.5. Safety and Fail-Safe Design Considerations

For a successful transition from laboratory development to clinical application, several key aspects related to regulation, safety, and integration into rehabilitation practice must be considered.

When developing an exoskeleton and assembling a prototype, it is necessary to ensure that the biomechanical and electromechanical parameters of the system are consistent with the acceptable natural range of human movement. This reduces the load on soft tissues and ensures user comfort during rehabilitation. Another key requirement is to ensure user safety when using the device in both laboratory and clinical settings. The comprehensive safety measures taken must comply with the following standards:

ISO 13482:2014—Robots and robotic devices—Safety requirements for personal care robots [58].

ISO 14971:2019—Medical devices—Application of risk management to medical devices [59].

IEC 80601-2-78:2019—Medical electrical equipment—Part 2–78: Particular requirements for basic safety and essential performance of medical robots for rehabilitation, assessment, compensation or alleviation [60].

IEC 60601-1:2020—Medical electrical equipment—Part 1: General requirements for basic safety and essential performance [61].

When developing an ankle joint exoskeleton with a remote control module, the following safety criteria must be taken into account:

1. Design requirements include basic measures to protect against mechanical injuries during movement. To do this, the exoskeleton must be securely fixed to the limb. At the same time, the weight of the exoskeleton must be minimized to prevent muscle fatigue in the user and increase the duration of safe rehabilitation sessions. A system to protect against falls and impacts must also be provided.

2. Electromechanical safety must include protection against overheating of motors and electronic components. These measures eliminate the possibility of electric shock when the user interacts with the device. A reliable actuator control system and backup power supply for emergency shutdown of the device must also be provided.

3. The exoskeleton control system must provide protection against accidental commands and a safe emergency stop mechanism. Data transmission channels must be encrypted for remotely located mechanisms, as well as for backup control channels. The main requirement is to limit the speed and range of movement within acceptable parameters. A technical condition monitoring system should be provided to track key parameters.

4. The materials used in the construction of the exoskeleton should be biocompatible, corrosion-resistant, and hygienic on surfaces that come into contact with the user’s skin. It is recommended to use durable, non-toxic, and easily cleanable materials to prevent irritation, allergic reactions, and microbial contamination during prolonged use of the device. Surfaces that come into contact with the skin should have a smooth coating that prevents the formation of burrs and microcracks and allows for regular disinfection without compromising mechanical properties.

5. The developed system should provide for integration into existing clinical protocols for lower limb rehabilitation and support for personalized adaptive modes. In this case, movement parameters are recorded using correctly configured operating modes and monitoring of therapy effectiveness. This makes it possible to adjust protocols to the individual needs of the patient.

In the experimental study, one of the design elements of safety is the use of a screw mechanism with a self-locking effect. It provides passive fixation in the selected position in the event of power loss or failure. The main advantage of the screw transmission is the prevention of sudden jerks, smoothly converting rotational movements into linear ones. This allows the exoskeleton to hold the limb in the desired position without continuous use of the stepper motor, which facilitates rehabilitation and reduces the load on the drives. The high precision and stability of the screw mechanism at low energy consumption reduces the risk of re-injury and contributes to the effective recovery of the patient.

To accurately and safely control the movement of the exoskeleton actuator, it is necessary to ensure consistency between the biomechanical and electromechanical parameters of the exoskeleton prototype. Since the physiological range of motion of the ankle is measured in degrees, it is necessary to reproduce the conversion of angular displacement into stepper motor control pulses (steps). Considering these features, according to the initial data of the main steam meters of the NEMA 17 stepper motor, the following values were taken:

1. One complete revolution (360°) is equivalent to 200 steps of the stepper motor, taking into account the conversion to linear displacement by 8 mm. Then one control pulse (step) for the stepper motor will be equal to:

(1) $$\delta_{step}=\frac{360^{{}^{\circ}}}{200}{=1.8}^{{}^{\circ}}$$

2. Therefore, a linear displacement of 1° of rotation would be equal to:

(2) $$l=\frac{8}{360^{{}^{\circ}}}=0.0222\;\mathrm{mm/deg.}$$

3. The number of steps per 1 mm of displacement is determined by:

(3) $$\xi_{step}=\frac{200}{8}=25\;\mathrm{step/mm.}$$

4. The conversion of a given angle to millimeters is determined by:

(4) $$x_{step}=\alpha_{deg}\times l=\alpha_{deg}\times 0.0222\;\mathrm{mm}$$

$\alpha_{deg}$—required specified rotation angle in degrees.

5. Conversion of angular value to stepper motor steps is determined by:

(5) $$S_{step}=\xi_{step}\times x_{step}=\alpha_{deg}\times 0.0222\times 25=\alpha_{deg}\times 0.555$$

The calculations allow us to determine the number of pulses required for the actuator to achieve the specified angle of rotation of the prototype exoskeleton’s foot, assuming that one full rotation is converted into a linear displacement of 8 mm.

These calculations are necessary to ensure coordinated interaction between the biomechanical and electromechanical systems, facilitating precise control of the exoskeleton prototype. Direct conversion of angles into the number of steps allows the motor to move the mechanical part to the required angle, generating the correct control signals for the exoskeleton during rehabilitation.

**Figure 9:**
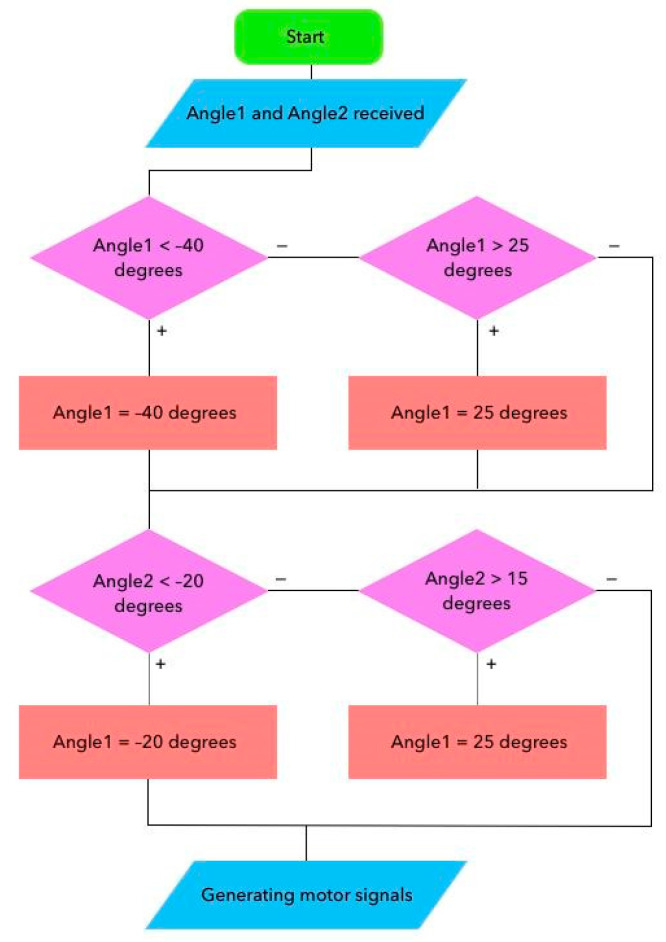
Flowchart for normalizing tilt angles and generating exoskeleton control signals.

For a continuous update process and stable transmission of control data in real time, a cyclic control algorithm is implemented in Figure 9. It is based on step-by-step normalization of the angles of inclination and their conversion into control pulses for the stepper motor.

The values of the Angle1 angles correspond to the movements of Dorsiflexion, Plantar flexion, and Angle2 to the movements of inversion and eversion. During initialization, the system starts and receives angle values from the Remote control device, which are converted into control pulses (steps) for the stepper motor. Before sending the motion control signal to the actuator of the prototype exoskeleton, the Angle1 and Angle2 limits corresponding to the RoM are checked. For Angle1, the system will limit the values in Dorsiflexion to 25°, Plantar flexion is reached to −40°, respectively. For Angle2, the maximum limits in inversion are −20° and eversion to 15°. When the specified value is exceeded, the angles are adjusted and then converted into control signals for stepper motors, and a command is sent to the actuator of the prototype exoskeleton. This ensures safe movement of the exoskeleton foot within the physiological range, preventing overexertion and risks of ankle injury during rehabilitation. This approach makes it possible to avoid parameters going beyond physiologically acceptable or mechanically safe ranges, and it has the ability to adapt to the individual and age-related characteristics of the rehabilitated patient.

To ensure safe and stable operation of the drive modules, the phase current setting on the TB6600 drivers was set at 1.2 A, which corresponds to the nominal parameters of NEMA 17 stepper motors and provides a torque that does not exceed the safe range for the ankle joint (up to 10 N·m). This mode prevents overheating and mechanical overload of components during laboratory tests without patient involvement.

The measures listed are aimed at assessing safety, comfort, the effectiveness of rehabilitation exercises, and compatibility with existing rehabilitation methods.

## 4. Results and Discussion

The structure of the experimental exoskeleton prototype was designed in SolidWorks Simulation Professional (2021) and manufactured using FDM printing from PLA filament. PLA was chosen at an early stage due to the requirements of laboratory validation and functional testing. The material is inexpensive, technologically suitable for rapid prototyping, and allows for quick verification of design solutions. The prototype, with a total weight of 3 kg, is intended solely for concept verification and is not intended for clinical use. In subsequent design stages, when moving on to the creation of a prototype, more reliable materials will be used, such as Carbon Fiber-Reinforced Plastics (CFRP), Nylon (PA, Nylon), and Thermoplastic polyurethane (TPU). These materials meet the requirements for durability and biomedical use, which are compatible with sterilization procedures in accordance with ISO 10993 [62] and ISO 17664 [63]. Therefore, all experiments presented in this work were performed under laboratory conditions without human participation, focusing on functional and communication validation of the developed mechatronic module.

The prototype assembly process combines mechanical and electronic components: the main body, Arduino board Mega 2560, Actuator Mini Electric 12 V, Servomotor NEMA 17, Driver TB6600, MPU-6050.

**Figure 10:**
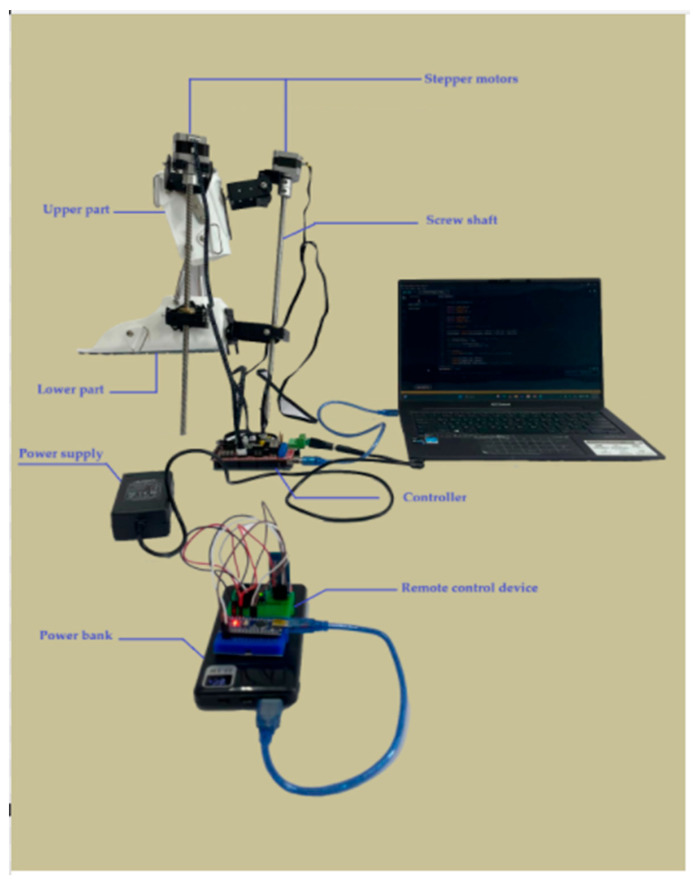
Experimental ankle exoskeleton module integrated with the main components of the wireless platform.

To test the functionality and compliance with biomechanical requirements in the sagittal and transverse planes, the exoskeleton prototype was integrated with a wireless communication platform. The experimental stand of the ankle joint exoskeleton prototype with the main components of the wireless communication platform is shown in Figure 10.

**Figure 11:**
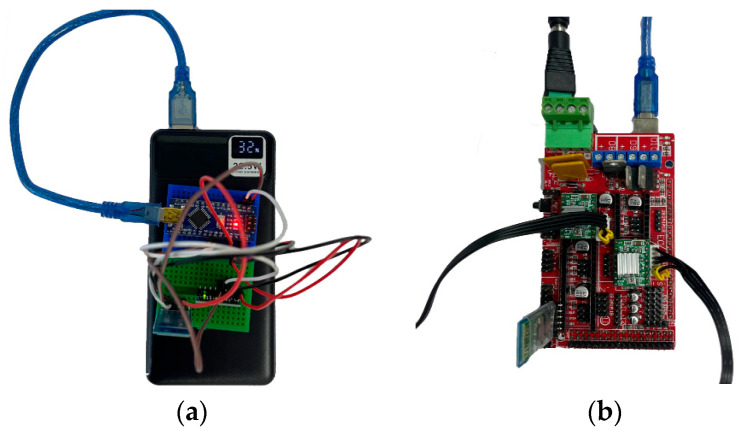
Wireless communication platform of the ankle exoskeleton prototype: Remote control device (**a**), exoskeleton executive unit (**b**).

The wireless communication platform shown in Figure 11 consists of a remote control device unit and an exoskeleton actuator unit.

Functional testing of the exoskeleton prototype is carried out without wired connections (operating frequency 2.4 GHz, range up to 10 m). If the wireless connection is lost due to exceeding the permissible range or interference, the receiving controller stops receiving and processing commands. In this case, a new trajectory is not initiated. The position of the node is passively held by a self-locking screw mechanism, and exceeding physiological limits is prevented by the RoM restriction algorithm on the receiver side.

### Experimental Study of an Ankle Exoskeleton Prototype with Integrated Wireless Communication Platform

In accordance with biomechanical characteristics, the maximum limits of the restricted range of motion (RoM) of the ankle joint were set in the control algorithm based on the data in Table 2. These values reflect physiologically justified limits of ankle joint movement, ensuring safe operation of the integrated system during experimental testing.

The first part of the experimental study focused on analyzing the movements of the exoskeleton in the sagittal plane, along which dorsal and plantar flexion of the ankle joint occur. In this case, the starting position is a neutral position in accordance with the natural vertical orientation of the foot at an angle of 90° to the lower leg. This position was used as the switching point for the directions of dorsiflexion and plantar flexion movements.

**Figure 12:**
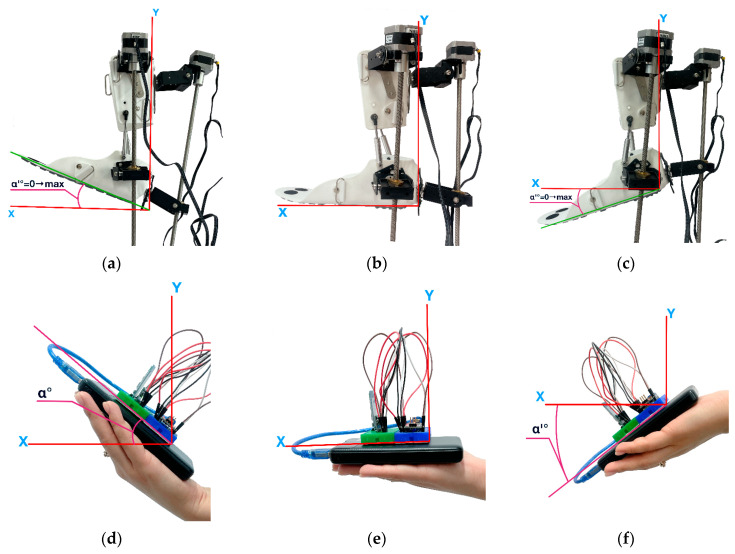
Positions of the exoskeleton prototype and Remote control device in the sagittal plane along the *X* axis at different angles: Dorsiflexion (**a**), Neutral position (**b**), Plantar flexion (**c**), and (**d**–**f**) positions of the Remote control device.

The moment of deactivation of control pulses to the exoskeleton’s actuators for switching is set within a range of 1 to 3 s. In accordance with the concept of an integrated wireless communication platform, the exoskeleton’s position is controlled remotely. The Arduino Nano controller located in the remote control device collects and processes data on movement and orientation using the MPU-6050 sensor. Then, using the HC-05 Bluetooth module, it transmits the control signal to the main Arduino Mega controller. After processing the data received from the Arduino Nano and checking it for compliance with the specified RoM limits, the NEMA 17 stepper motors are controlled via the RAMPS 1.4 expansion board. When the spatial position of the controller in the remote control device changes, in this case, tilting forward and backward, the exoskeleton prototype must respond accordingly. The results of the experimental studies are shown in Figure 12.

In the neutral position, no control signals are sent from the remote control device (Figure 12e) to the exoskeleton prototype, and its angle of deflection relative to the *X*-axis remains at α = 0°. Accordingly, in this case, the prototype also assumes a neutral position (Figure 12b). In this state, there will be no movement activation, fixing the resting position of the foot. At this point, movement stabilization and switching between the dorsiflexion and plantar flexion phases are ensured. When a control signal is applied to lift upwards (Figure 12d), the position of the exoskeleton prototype (Figure 12a) is raised to α = 25° and returns to its original neutral position. After stabilizing the position from the neutral point, the remote control device switches to plantar flexion (Figure 12f), where the position of the prototype switches in the opposite direction (Figure 12c) and descends to α’ = 40° and returns to its original position. Control commands generated based on changes in the tilt of the remote control device along the *X*-axis ensure smooth and coordinated kinematics of the exoskeleton prototype movement, corresponding to the biomechanics of the ankle joint.

**Figure 13:**
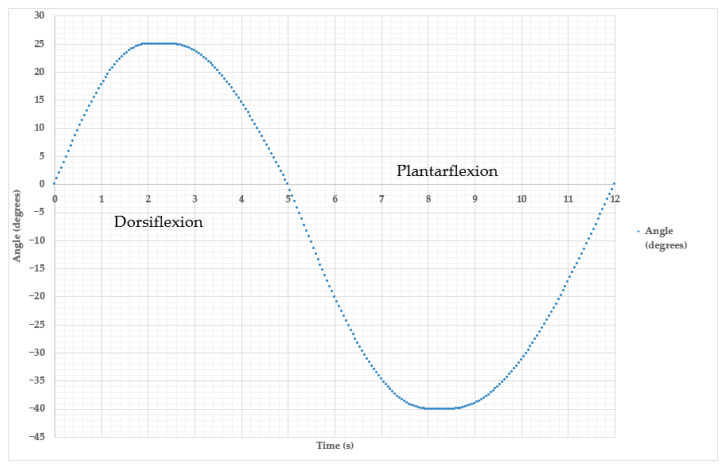
Graph of change in the angle of motion of the exoskeleton prototype in the cycle of dorsiflexion and plantarflexion for 12 s.

The results of the continuous cyclic movement process of the exoskeleton prototype, consisting of two main phases, dorsiflexion and plantar flexion, are shown in Figure 13.

The full movement cycle took 12 s in the working range for dorsiflexion 25° and plantar flexion 40°. The lifting of the exoskeleton foot towards the lower leg (0–5 s) covers a range of motion from 0° to 25°, which corresponds to the limitations (Table 2) in the dorsiflexion phase. The maximum value is reached at approximately 2.5 s, and by gradually reducing the angle of inclination of the exoskeleton prototype, the transition to the plantar flexion phase (5.25–12 s) occurs. In this case, the angle of deflection gradually decreases from 0° to 40°, which corresponds to the exoskeleton foot being released downwards away from the shin. Maximum plantar flexion is recorded at around 9 s, after which the foot returns to a neutral position at 12 s. The intended full cycle can be repeated depending on the calibration for training patients with various musculoskeletal disorders to walk. When the RoM is exceeded, the exoskeleton blocks the movement, preventing commands from the remote control device that exceed the set limits from being executed. This ensures user safety and prevents excessive stress on the ankle joint during rehabilitation.

**Figure 14:**
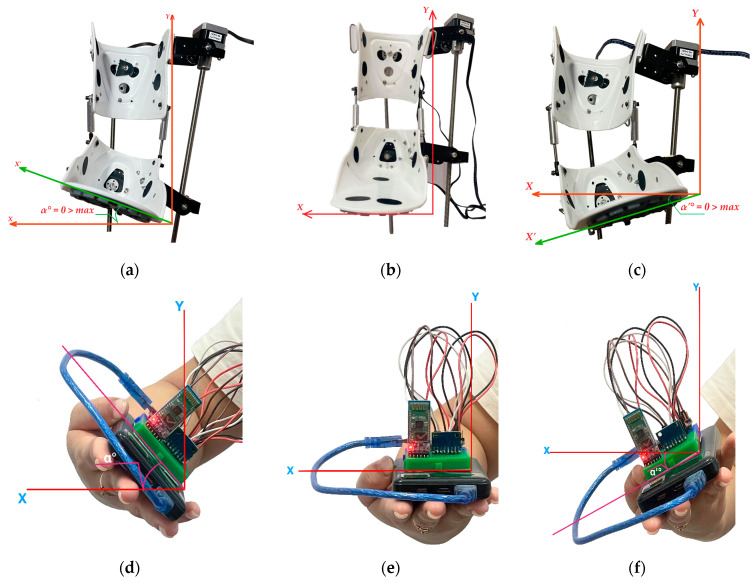
Positions of the exoskeleton prototype and Remote control device along the transverse plane of the *Y*-axis at different angles: Eversion (**a**), Neutral position (**b**), Inversion (**c**), and (**d**–**f**) positions of the Remote control device.

The second part of the experimental study was devoted to analyzing the movements of the exoskeleton in the transverse plane of the *Y*-axis. Inversion and eversion movements of the foot are performed along this axis. The results of the experimental studies are shown in Figure 14.

**Figure 15:**
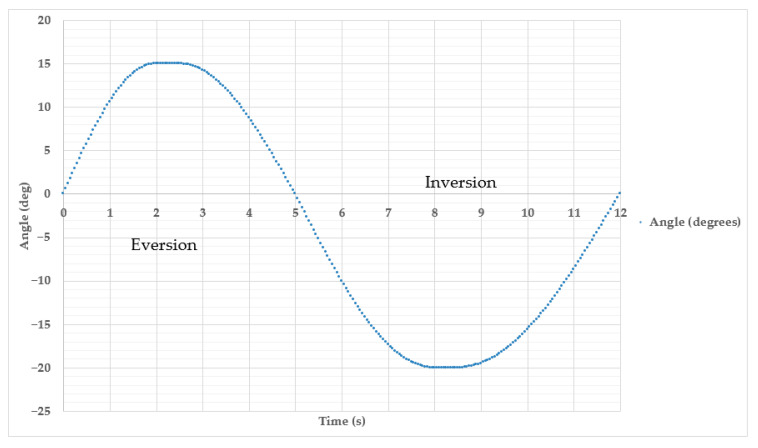
Graph of change in the angle of motion of the exoskeleton prototype in the Eversion and Inversion cycle for 12 s.

In Figure 14e, the remote control device system is in a state of equilibrium where the angle of position deviation relative to the *Y*-axis is α = 0°. Due to the lack of applied control signals, the prototype exoskeleton (Figure 14b) will have the same position where lateral movement stability is ensured. This resting state serves as a reference point for switching between inversion and eversion, which means no inward (inversion) or outward (eversion) rotation of the foot. The position of maximum eversion is achieved by tilting the remote control device to the right side (Figure 14d). In this case, the exoskeleton foot deviates outwards to α = 15° (Figure 14a) and returns to the neutral position. The command to tilt the Remote control device to the left side (Figure 14f) corresponds to the Inversion movement, where the exoskeleton foot is tilted inwards (Figure 14c) by up to α’ = 20° and returns to the initial state to stabilize in the neutral position. The results of a full Eversion and Inversion cycle consisting of 12 s are presented in Figure 15.

Between 0 and 5.25 s, the Eversion phase is realized, which corresponds to a deviation of the outer edge of the foot from the midline of the tibia between 0° and 15°. The angle of deviation decreases successively in the Inversion phase to 20° in the period 5.25–12 s. This type of movement is important in analyzing lateral stability of the foot and is often used in rehabilitation after ankle ligament sprains or in the diagnosis of gait disorders. If RoM is exceeded, a restriction in the exoskeleton prototype’s actuator unit is activated, blocking further movement beyond the set amplitude in the Remote control device.

The results demonstrate the smooth and controlled nature of cyclic activity, similar to physiological ankle joint motion. Cyclic smooth movements may be useful for walking training as well as biomechanically controlled exoskeletons, where accurate imitation of physiological joint movement is required.

Experimental studies confirmed the functionality of the established system of a wireless communication platform transmitting control signals via Bluetooth modules (HC-05). The consistency of the Remote control device with the prototype ankle exoskeleton was also demonstrated. Changes in positions in the Remote control device contributed to the reproduction of corresponding smooth movements in the prototype within the set range.

As part of functional testing, the accuracy and delay parameters of the remote control system were quantified. The results are shown in Table 3.

To evaluate measurement consistency, each test was repeated ten times under identical conditions. The results showed a repeatability coefficient of 0.91, indicating stable and reproducible system performance. The mean RMSE values were 23.9° ± 2.6° (SD) in dorsiflexion/plantarflexion and 12.8° ± 1.9° (SD) in inversion/eversion. However, such deviations are relatively high for ankle rehabilitation. In clinical applications, the permissible angle-tracking error should be considerably smaller, typically ≤5–10° in the sagittal plane and ≤5–8° in the frontal plane, with latency below 50–80 ms and packet loss under 0.1% to ensure safe and repeatable motion [25,36,39,41,43,45].

The increased RMSE values are mainly due to drift of the IMU sensor and inclination conversion errors, lack of feedback in the stepper motor control loop, as well as elasticity and backlash in the screw gear mechanism made of polymer materials. Despite the stable and synchronized operation of remote control in real time, this prototype should be considered as a laboratory platform for checking the functionality and consistency of remote control modules.

Further studies plan to implement closed-loop control systems and improved sensor modules to reduce tracking errors and delays to levels that meet clinical requirements.

## 5. Conclusions

This article presents the results of an experimental study and laboratory evaluation of a mobile prototype of an ankle exoskeleton with a remote control module based on the Bluetooth wireless platform. The architecture of the system is implemented in the form of a two-level configuration “transmitter-receiver,” which provides the transmission of control signals in real time between the remote control and the actuator module. To implement functional testing of the device, the relevance of the problem was substantiated, as well as existing solutions in the field of innovative developments in the field of rehabilitation of the lower extremities were studied. A general concept was developed and maximum limits of limited ankle range of motion (RoM) were determined to validate the proposed system. The structure of the experimental exoskeleton prototype was designed in SolidWorks Simulation Professional (2021) and manufactured using PLA filament FDM printing. The basic structure of the experimental exoskeleton prototype combines mechanical and electronic components such as the base case, Arduino Mega 2560 board, Mini Electric 12 V drive, NEMA 17 servomotor, TB6600 driver and IMU MPU-6050. Its integration with a wireless communication platform based on Bluetooth modules (HC-05) provided stable control of the exoskeleton foot movement in real time, both in the sagittal (Dorsiflexion/Plantar Flexion) and transverse (Inversion/Eversion) planes. At the same time, the exoskeleton foot movement control system was synchronized with the spatial position remote control device, where the main movements in different planes were performed within the specified range.

Experimental tests conducted in laboratory conditions before clinical validation confirmed the stable and reproducible operation of the system (repeatability coefficient 0.91). The root mean square error (RMSE) was 23.9° ± 2.6° for dorsal/plantar flexion and 12.8° ± 1.9° for inversion/eversion. The average signal transmission delay is about 100 ms, packet loss is less than 0.6%. These data confirm the functional integrity of the system and the ability of the exoskeleton to follow given trajectories in real time. The developed prototype with a mass of about 3 kg provides a torque of 8–10 N·m, sufficient for basic movements of the ankle joint. The design is focused on mobility, modularity and safety. Self-braking screw gear provides position retention when the signal is lost, and RoM and current limiting algorithms prevent physiological angles from being exceeded.

Further research will focus on implementing feedback control, integrating angle sensors, improving accuracy, improving ergonomics, and conducting clinical validation with human involvement.

## Figures and Tables

**Table 1:** Comparative technical parameters and areas of application of existing robotic systems for lower limb rehabilitation.

Device	Weight (kg)	Actuation Type	DoF	Max. Torque (N·m)	Range of Motion (°)	Control Method	Application	Features	Limitations
Lokomat (Hocoma, Switzerland) [18]	≈1000	Electric actuators, cable belt, treadmill	4 (hip, knee)		0–45° (hip), 0–60° (knee)	Gait imitation, position control	Clinical rehabilitation and gait restoration for patients with spinal cord injury, stroke, traumatic brain injury, and neurological disorders	High efficiency in clinical use	Bulky, expensive
AnkleBot (MIT, USA) [20]	3.5–4	Electric actuators	2	≈12	±25° PF/DF, ±15° INV/EV	PID/impedance control	Local ankle joint therapy, motor learning, neuroplasticity enhancement	Based on motor learning principles and repetitive motion exercises for restoring motor control	Stationary, not portable
ReWalk (ReWalk Robotics) [21]	20–25	Electric actuators	4	40–60 H·м	0–120° hip, 0–90° knee	Inertial sensors, manual input	Home and ambulatory rehabilitation	Enables upright mobility and walking with crutches, reducing risks of sedentary lifestyle complications	Requires crutches, high cost
Single-Legged Rehabilitation Exoskeleton [24]	≈6–7	Electric (BLDC) actuators	2 (hip, knee)	18.1 H·м, 2.6 H·м	45° hip, 60° knee	Wireless PID, Bluetooth/IMU	Tele-rehabilitation, outpatient gait recovery	Used for gait evaluation and motion training via IMU and Bluetooth	Requires clinical validation
A Lower-Body Exoskeleton Platform [25]	≈10	Cable drive (Bowden), AC motors (400 W)	4 (hip, knee)		Up to anatomical limit	CANopen, distributed control	Experimental gait restoration testing	Simulates gait motion for post-injury rehabilitation	Cable losses, not suitable for home rehabilitation
Medical Exoskeleton “Remotion” [26]	≈15	Electric (BLDC) actuators	4			Wi-Fi remote control, EMG/FES	Stationary clinical rehabilitation, motor re-learning	Functional electrical stimulation and gait recovery under remote supervision	Heavy, complex gait dynamics

**Table 2:** Maximum limits of the limited range of motion of the ankle joint (RoM).

№	X and Y Movements	RoM
1	Plantar flexion	40°
2	Dorsiflexion	25°
3	Inversion (inward rotation)	20°
4	Eversion (outward rotation)	15°

**Table 3:** Quantitative performance indicators of a prototype remote-controlled ankle exoskeleton.

Parameter	Value	Description
RMSE dorsiflexion/plantar flexion	23.9°	Root mean square tracking error of the joint angle
RMSE inversion/eversion	12.8°	Root mean square error along the frontal axis
Amplitude dorsiflexion/plantar flexion	65.0°	Motion range in the sagittal plane
Amplitude inversion/eversion	35.0°	Motion range in the frontal plane
Repeatability dorsiflexion/plantar flexion	63.3%	Stability of motion across cycles
Repeatability inversion/eversion	63.5%	Consistency of movements between cycles
Average communication latency	100 мc	Typical delay for the Bluetooth HC-05 module
Average power consumption	5.2 Bт	Power during active operation of stepper motors
Bluetooth packet loss	0.6%	Reliability of the wireless communication link

## Data Availability

The data presented in this study are available on request from the corresponding author.
